# High Expression of COL17A1 Predicts Poor Prognosis and Promotes the Tumor Progression via NF-*κ*B Pathway in Pancreatic Adenocarcinoma

**DOI:** 10.1155/2020/8868245

**Published:** 2020-12-16

**Authors:** Feiyu Mao, Duguang Li, Zechang Xin, Yan Du, Xiaodong Wang, Peng Xu, Zhennan Li, Jianjun Qian, Jie Yao

**Affiliations:** ^1^Clinical Medical College of Yangzhou University, Yangzhou, Jiangsu 225001, China; ^2^Department of General Surgery, Sir Run Run Shaw Hospital, School of Medicine, Zhejiang University, Hangzhou, China; ^3^The First Affiliated Hospital of Dalian Medical University, Dalian, Liaoning 116044, China; ^4^Department of Hepatobiliary and Pancreatic Surgery, Northern Jiangsu People's Hospital, Nantong Western Road, Guangling Qu, Yangzhou, Jiangsu 225001, China

## Abstract

COL17A1 (collagen type XVII alpha 1 chain) is known to be upregulated and has a prognostic role in many malignancies, as well as contributing to cell proliferation, apoptosis, and invasion. However, little knowledge is available on the expression and prognostic value of COL17A1 in pancreatic adenocarcinoma (PDAC). In our study, we searched the public database and found that mRNA and protein levels of COL17A1 are commonly upregulated in PDAC tissues. The immunohistochemical analysis conducted by us revealed enhanced expression of COL17A1 protein in 169 PDAC samples compared with that in 67 adjacent normal tissues. We also observed a significantly positive correlation between COL17A1 expression and lymph node metastasis (*p* < 0.0001), TNM clinical stage (*p* < 0.0001), and pathology differentiation (*p* < 0.01). The KM-plot results indicated that PDAC patients with a high COL17A1 expression have a poorer overall survival (*p* < 0.001) than those with a low COL17A1 expression. The result of the Cox regression analysis of multivariate data suggested COL17A1 is an independent prognostic indicator of PDAC patients' overall survival. CCK-8, wound healing, and transwell assays suggested that COL17A1 knockdown markedly inhibited tumor proliferation and invasion in PDAC cells, and cells with COL17A1 overexpression had a prominently higher proliferative and invasive capacity. Knockdown of COL17A1 significantly upregulated the apoptosis rate. We deduce that upregulated COL17A1 activated the NF-*κ*B pathway in PDAC cells. In summary, our studies showed the prognostic value of COL17A1 in PDAC and that COL17A1 may act as a molecular therapeutic target for PDAC treatment.

## 1. Introduction

Pancreatic adenocarcinoma is known for its late-stage diagnosis, aggressive growth, and poor clinical benefit in terms of treatment strategies [1, 2]. At the same time, a patients' one-year survival rate is 19–34% for all stages of the disease and 4–11% at 5 years because of the high metastatic and recurrent potential [3, 4]. Clinicopathologic parameters, like the TNM system, pathological classification, and CA19-9 level, have been widely used for prognostic evaluation, but most of them do not fully predict individual clinical outcomes [5, 6]. Until now, researchers have discovered about 12 signaling pathways, abnormal multioncogene activation, or abnormal tumor suppressor gene inactivation that play a part in the growth and acceleration of pancreatic adenocarcinoma [7, 8]. Therefore, to find potential molecular markers and therapeutic targets, unveiling potential molecular mechanism of pancreatic adenocarcinoma has important clinical significance for improving the diagnosis and treatment of the disease.

Upregulated extracellular matrix proteins, like collagens, can promote the first steps of tumor cell metastasis, which involves invasion into the surrounding tissue and infiltration into the bloodstream in the tumor microenvironment [9]. COL17 (collagen XVII), a transmembrane protein, which is nonfibril forming, is encoded by COL17A1 and is also referred to by the names BP180, ERED, and BPA-2 [10]. COL17A1 is a vital component of mature type I HDs (hemidesmosomes) located in a region at 10q25.1 and has dual roles, as a molecule that adheres to the cell-matrix and a surface receptor for the cell [10]. Furthermore, type-1 HDs disassembly in pancreatic adenocarcinoma has been shown to be associated with COL17 cleavage and may promote pancreatic cancer cell migration and invasion [11]. The onset of autoimmunity to COL17 and COL17A1 mutations cause the epidermis and the membrane beneath it to lose adhesion, thereby leading to blister formation and skin diseases [12, 13]. Mutations in COL17A1 may be the leading cause of both generalized atrophic benign and junctional epidermolysis bullosa [14]. COL17 has two homotrimeric forms: a transmembrane protein of full-length and another soluble form called ectodomain, or LAD-1, is the proteolytically processed full-length form [15, 16]. Several studies have documented that type I hemidesmosomes that occur in regular epithelial cells were untraceable in breast cancer cells [17] and pancreatic ductal epithelium [18]. COL17 also expresses highly in normal epithelial tissues, like in the skin, mammary gland, skin, and colon [19]. In epigenetic studies, methylation of COL17A1 promoter can be used to predict its misexpression, patient outcome, and increased invasion in epithelial cancers [9]. Another study has reported a strong correlation between the progression of the tumor and metastasis with enhanced COL17 expression, which was associated significantly with infiltrative growth, distant metastasis, and poor outcome in colorectal carcinoma [20]. A recent report suggests that COL17A1 is a p53 transcriptional target gene and inhibits the metastasis and invasion of breast cancer [21]. However, the expression and biological significance of COL17A1 in pancreatic adenocarcinoma has not been reported yet.

Therefore, we analyzed the expression level of COL17A1 in PDAC tissues. Furthermore, we explored the correlation between COL17A1 expression and clinicopathological characteristics in pancreatic cancer and observed that the overexpression of COL17A1 in pancreatic adenocarcinoma patients is significantly correlated with overall survival (OS). We also found that COL17A1 may promote the proliferative and invasive phenotype of PDAC cells by inactivating the NF-*κ*B (nuclear factor-kappa B) pathway.

## 2. Materials and Methods

### 2.1. Patients and Tissue Specimens

All the tissues of pancreatic adenocarcinoma were obtained from the patients who had surgical abscission treatment from October 2015 to October 2019 in Northern Jiangsu People's Hospital. The aforementioned patients had not undergone chemotherapy or radiation therapy prior to the surgical procedure and were diagnosed independently by two pathologists. One sixty-nine cancerous and 67 noncancerous paraffin-embedded specimens were used for immunohistochemistry. The Cancer Genome Atlas (TCGA) confirmed the differential expression of COL17A1 as well as the Gene Expression Omnibus (GEO) database (GSE62452, GSE28735, GSE15471, GSE62165). The written consent of all the patients was obtained and the study was approved by the Northern Jiangsu People's Hospital Ethics Committee.

### 2.2. Immunohistochemistry

The sample was blocked and incubated with the COL17A1 Ab (1 : 100) for 2 h at 22°C, and an HRP conjugated goat anti-rabbit Ab was used as the secondary antibody. Two pathologists, blinded for patients' clinical data independently evaluated the immunohistochemical staining. COL17A1 expression levels were classified by the semiquantitative method that combines the intensity of the staining as well as the percentage of cells that stained positive. The percentage of positive PDAC cells was graded on the following five levels of scores: none: 0; 1–25%: 1; 26–50%: 2; 51–75%: 3; and 76–100%: 4. The staining intensity was graded on the following four levels of the scores as follows: none: 0, weak: 1, moderate: 2, and strong: 3. The low- or high-expression group was decided by the total score which was determined by the percent of positive cell score × staining intensity score (total score > 6: high expression, score ≤ 6: low expression) [18, 22].

### 2.3. Cell Lines and Their Transfection

Shanghai Institute of Nutrition and Health in Shanghai, China, provided the human PDAC cell lines PANC-1, SW1990, AsPC-1, and HPAF-II. The cells were cultured in a medium with streptomycin-penicillin (1%) along with fetal bovine serum (10%) and incubated at a temperature of 37°C and 5% carbon dioxide-fed humidified incubator. As per the manufacturer's directions, LipoPlus (Invitrogen, USA) was used for transfection. COL17A1-siRNA (small interfering RNAs) and negative control were synthesized by GenePharma Technology (Shanghai, China), and the overexpressing plasmids COL17A1 were purchased from Hanyin Biotechnology (Shanghai, China).

### 2.4. Real-Time Quantitative PCR

The Trizol reagent from Invitrogen (China) was used to extract the total RNA while the RT-PCR Kit sourced from Vazyme (China) was used to reverse transcribe the samples. The primers used in this study were COL17A1: forward: 5′‐GCAGAGCTGAGTAGTCGCAT‐3′; reverse: 5′‐AATTCAGACCCTCGCAGCAA‐3′; C3H6O3 (Glyceraldehyde)‐3‐G6PD_N (phosphate dehydrogenase): forward: 5′‐GAAGGTGAAGGTCGGAGTC‐3′; reverse: 5′‐GAAGATGGTGATGGGATTTC‐3′. Each Ct value was normalized to GAPDH Ct to determine relative expressions. RT‐qPCRs were performed according to the MIQE (Minimum Information for Publication of Quantitative Real-Time PCR Experiments) guidelines [23].

### 2.5. Western Blot Assay

RIPA buffer (Solarbio, China), containing protease inhibitor was used to lyse the cells and was maintained on ice for 30 min. After transferring the resolved protein from the polyacrylamide gel onto a membrane, 5% mixture of bovine serum albumin from Invitrogen (China) was used to block the membranes and incubation was done overnight at 4°C. Subsequently, for a duration of 1 h, incubation was conducted using horseradish peroxidase (HRP) conjugated goat anti-mouse or goat anti-rabbit antibody sourced from Cell Signaling Technology, China. Anti-p-p65, anti-p-I*κ*B*α*, anti-COL17A1, I*κ*B*α*, and anti-p65 antibodies sourced from Cell Signaling Technology were used to perform western blot assay and anti-GAPDH antibody (Abcam) was the loading control. Image *J* was used for calculating the protein expression.

### 2.6. Transwell and CCK-8 Assays

The incubation was conducted at 24, 48, and 72 h at 37°C. Plates (96-well) were seeded at 5000 cells/well, and then after incubating at 37°C for 2 h, 10 *µ*L from CCK-8 from Synthgene (China) was added. For all of the 24-well plates, prediluted Matrigel-coated transwell inserts were used and allowed to gel at 37°C for 30 min. Seeding of the cells was done at a density of 3 × 105 per insert and 500 *μ*L DMEM with 10% fetal bovine serum was mixed to transwell's lower chamber.

### 2.7. Detection of Apoptosis and Cell Cycle

Cells (5 × 10^5^) were harvested and placed in ice-cold ethanol (70%) for 12 to 24 h. Ribonuclease sourced from Thermo Fisher Scientific at a dosage of 10 mg/ml was added followed by 50 mg/ml of propidium iodide from Sigma and was incubated for 30 min in the dark at 37°C. Flow Cytometry from BD Bioscience (California) was used to analyze the cell cycle. FITC Annexin apoptosis kit from Vazyme (China) was used to analyze apoptosis by incubating the harvested treated cells with these reagents as per the manufacturer's protocol.

### 2.8. Tumorigenesis in Nude Mice

Four-to-six-week-old male nude mice (18–20 g) were obtained from the SLAC Laboratory Animal Co., Ltd. (Shanghai, China), and xenograft tumors were generated by using subcutaneous injections of 4 × 10^6^ cells. The mice were randomly categorized into the si-COL17A1 subcutaneous injection group and a control group with weekly twice injections. AsPC-1 cells were transduced with lentivirus vectors expressing COL17A1 siRNA. The nude mice were kept in pathogen-free specific conditions. In the course of experimentation on animals, the guidelines of China Animal Welfare Legislation strictly adhered at all times.

### 2.9. Statistical Analysis

Statistical analyses were carried out using the GraphPad Prism8 software and the SPSS Version 19.0. For real-time PCR, 2-ΔΔCt was calculated, and the inferential statistic Student's *t*-test was conducted to determine the *p*-value among the two groups. The log-rank and the Kaplan–Meier test were used to evaluate the survival curves. A *p* value < 0.05 was considered as statistically significant.

## 3. Results

### 3.1. COL17A1 Is Upregulated Significantly in Pancreatic Adenocarcinoma

**Figure 1:**
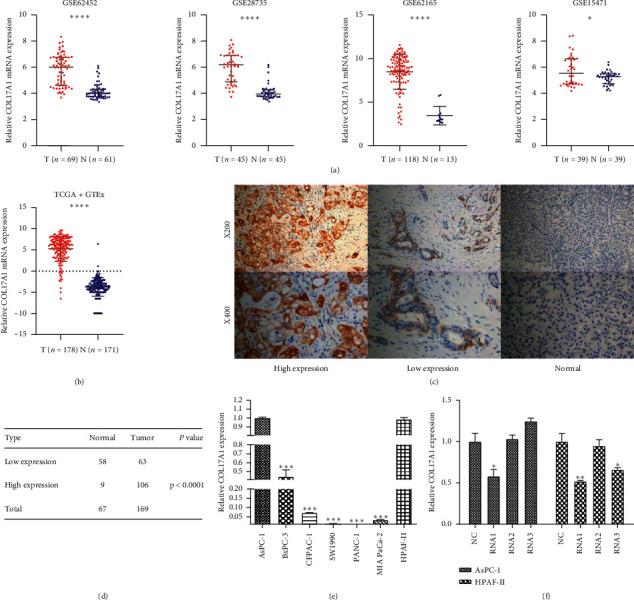
Differential expression between PDAC and normal tissues. (a) Differential expression of COL17A1 in GEO (GSE62452, GSE28735, GSE62165, and GSE15471) (RMA normalized gene expression values). (b) Differential expression of COL17A1 in TCGA and GTEx database by UCSC Xena visualization tool (normalized gene level expression value by log2(TPM+0.0001)). (c) Immunochemistry assay shows the high and low expression level of COL17A1 in PDAC and negative expression in adjacent normal tissues, shown at × 200 and × 400 magnification, respectively. (d) *χ*2 test was chosen for evaluating the differential expression between 169 PDAC and 67 adjacent normal tissues. (e) RT-qPCR shows the COL17A1 expression in six PDAC cell lines. (f) We selected the siRNA with the best knockdown effect from siRNA1, 2, and 3 according to RT-qPCR. RMA : robust multiarray average (^*∗*^*p* < 0.05, ^*∗∗*^*p* < 0.01, ^*∗∗∗*^*p* < 0.001, and ^*∗∗∗∗*^*p* < 0.0001).

Four microarray datasets from GEO (GSE62452, GSE28735, GSE15471, and GSE62165), TCGA, and GTEx database were analyzed to compute the differential expression of COL17A1 in human PDAC tissue [24–29]. The mRNA levels of expression of COL17A1 were upregulated in the tumor tissues when compared with the normal pancreatic tissue (Figures 1(a) and 1(b)). This is in accordance with the results of public database analysis, and immunohistochemical results show that the COL17A1 protein level was enhanced in tumor tissues in comparison with adjacent normal tissues (Figure 1(c)) at a significant level (*p* < 0.0001) (Figure 1(d)). These results clearly indicate that in PDAC tissues there was a noticeable increase in COL17A1, the mRNA as well as its protein levels. Further, the level of expression of COL17A1 was estimated in seven PDAC cell lines to select the suitable cells for further experiments (Figure 1(e)) and the siRNA with the best knockdown effect from siRNA1, 2, and 3 was chosen according to RT-qPCR (Figure 1(f)).

### 3.2. COL17A1 Is Significantly Associated with Lymph Node Metastasis, TNM Clinical Stage, and Pathology Differentiation in PDAC Patients

We used immunohistochemistry to assess the expression of COL17A1 protein in 169 paraffin-embedded pancreatic adenocarcinomas and 67 adjacent normal specimens. The immunostaining for COL17A1 was observed in the cytoplasm of PDAC cells, and 62.7% (106/169) of PDAC samples displayed high expression of COL17A1, while the other 37.3% (63/169) of samples showed low expression (Table 1). *χ* 2 test showed significant differential expression between PDAC and adjacent normal tissues (Figure 1(f)). Then, the relationship between COL17A1 protein level and the clinicopathologic parameters was analyzed.  Table 2 shows the correlation between the COL17A1 and clinicopathologic characteristics; the upregulation of COL17A1 was significantly associated with lymph node metastasis (N0 vs. N1-2; *p* < 0.0001), TNM clinical stage (I or II vs. III or IV; *p* < 0.0001) pathology differentiation (high or middle vs. low or no; *p* < 0.01). However, a negative relationship was found between the expression of COL17A1 and the other clinicopathologic factors, such as *T* classification, distant metastasis, gender, age, vascular invasion, and neural invasion.

### 3.3. Upregulated COL17A1 Predicts Poor Prognosis in PDAC Patients

**Figure 2:**
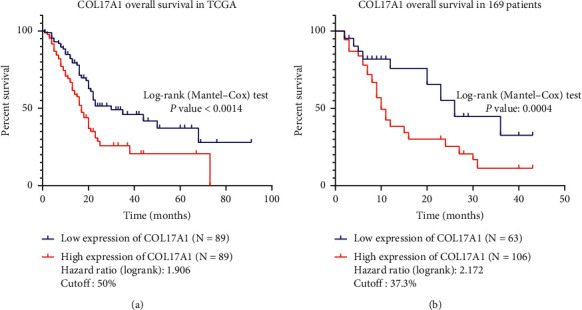
COL17A1 is significantly correlated with over survival in PDAC patients. (a, b) The Kaplan–Meier survival curves indicate that patients with high expression have a better overall survival time, respectively, from the TCGA database (*N* = 178, *p* < 0.01) and our 169 PDAC patients (*p* < 0.001).

We analyzed the relationship between COL17A1 expression and the OS time to evaluate the prognostic value of COL17A1 in PDAC according to the Kaplan–Meier survival curves and the log-rank test. Patients with high COL17A1 expression all had a significantly lesser OS than those with low expression in 178 (TCGA) and 169 (our cohort) PDAC patients (*p* < 0.01 and *p* < 0.001 resp., log-rank test) (Figures 2(a) and 2(b)).

In addition, we validated that COL17A1 features as an independent risk factor for patients' prognosis by univariate and multivariate analyses. Univariate Cox regression analysis indicated that age, clinical stage, *T* classification, metastasis, neural invasion, and the expression of COL17A1 were significantly associated with prognostic significance. In the multivariate Cox regression analysis, age, metastasis, neural invasion, and the expression of COL17A1 were independent prognostic predictors (Table 3) (*p* < 0.05, *p* < 0.05, *p* < 0.01, and *p* < 0.01, resp.). These findings showed that upregulated COL17A1 may be an independent prognosis factor in PDAC patients.

### 3.4. COL17A1 Promotes the Proliferation and Invasion of PDAC Cells In Vitro

**Figure 3:**
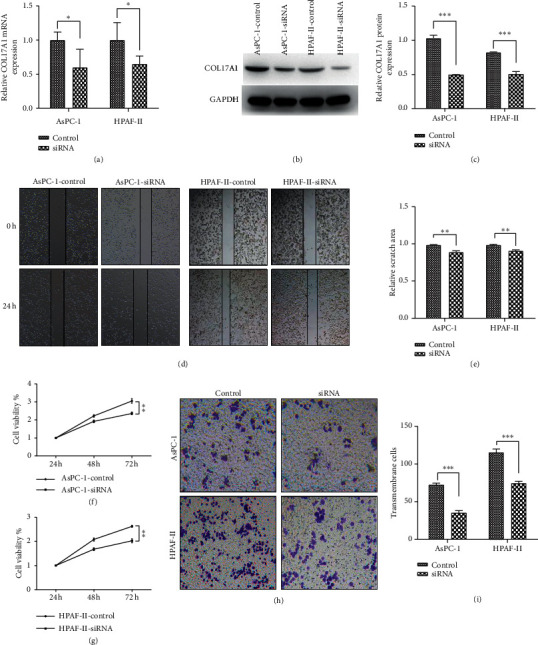
Downregulation of COL17A1 inhibited proliferation and invasion in PDAC cells. The verification of si-COL17A1 downregulation was evaluated by RT-qPCR (a) and western blotting in AsPC-1 and HPAF-II cells (b, c). Effects of COL17A1 downregulation on proliferation and migration were done by wound healing (d)-(e) and CCK-8 (f, g) assays. (h, i) Transwell assay measured the COL17A1 downregulation effects on the invasion. Results shown are the mean ± SD (^*∗*^*p* < 0.05, ^*∗∗*^*p* < 0.01, and ^*∗∗∗*^*p* < 0.001; the experiments were repeated at least three times). siRNA: transfected with small interfering RNAs.

**Figure 4:**
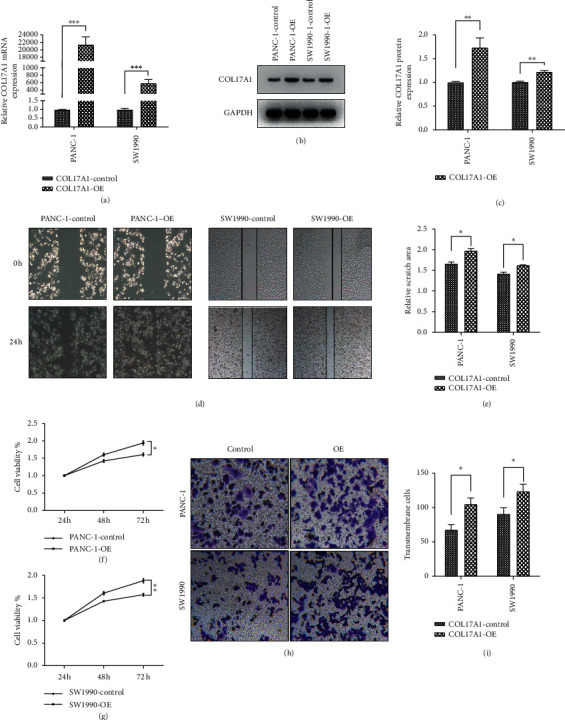
Upregulation of COL17A1 promoted the proliferation and invasion of PDAC cells. The verification of COL17A1 overexpression was evaluated by RT-qPCR (a) and western blotting in PACN-1 and SW1990 cells (b, c). Outcomes of overexpression of COL17A1 with regard to proliferation and migration were evaluated by wound healing (d, e) and CCK-8 (f, g) assays. (h, i) The transwell assay measured the outcomes of COL17A1 overexpression on the invasion. Results shown are the mean ± SD (^*∗*^*p* < 0.05, ^*∗∗*^*p* < 0.01, and ^*∗∗∗*^*p* < 0.001; the experiments were repeated at least three times). COL17A1-OE: transfected with COL17A1 overexpressing plasmids.

To test if COL17A1 promoted PDAC cell proliferation and invasion, we knocked down the expression of COL17A1 in AsPC-1 and HPAF-II cells with siRNA-COL17A1 and overexpressed COL17A1 in PANC-1, and SW1990 cells. The transfection efficiency was confirmed by RT-qPCR and western blot (Figures 3(a)–3(c) and Figures 4(a)–4(c)). Then, wound healing, CCK-8 assays, and transwell assays showed that the knockdown of COL17A1 notably inhibited the proliferation and invasion of AsPC-1 and HPAF-II cells compared to those in the control (Figures 3(d)–3(i)). While cells with COL17A1 overexpression had a prominently higher proliferative and invasive capacity than negative control in PANC-1 and SW1990 cells (Figures 4(d)–4(i)), these findings suggest that COL17A1 can enhance the proliferative and invasive ability of pancreatic adenocarcinoma cells.

### 3.5. COL17A1 May Promote PDAC Progression by Inactivating Cell Apoptosis

**Figure 5:**
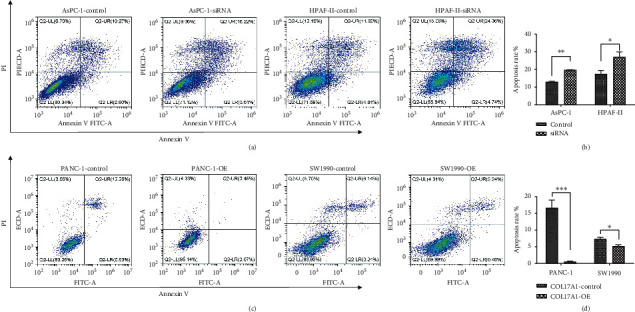
COL17A1 inhibited apoptosis of pancreatic adenocarcinoma. (a, b) Flow cytometric analysis was performed to evaluate cellular apoptosis using V/PI double staining annexin in COL17A1-siRNA cells. (c, d) Flow cytometric analysis was carried out to evaluate cellular apoptosis using V/PI double staining annexin in COL17A1-OE cells. Results shown are the mean ± SD (^*∗*^*p* < 0.05, ^*∗∗*^*p* < 0.01, and ^*∗∗∗*^*p* < 0.001). COL17A1-OE: transfected with COL17A1 overexpressing plasmids.

Cell apoptosis assay results show that knockdown of COL17A1 significantly upregulated the apoptosis level of AsPC-1 and HPAF-II cells compared with those in the control (Figures 5(a) and 5(b)). Further, COL17A1 overexpression cells had prominently lower apoptosis than negative control PANC-1 and SW1990 cells (Figures 5(c) and 5(d)). Therefore, COL17A1 may promote PDAC progression by inactivating cell apoptosis.

### 3.6. COL17A1 Promotes PDAC Progression by Activating NF-Kappa B Signaling Pathway

**Figure 6:**
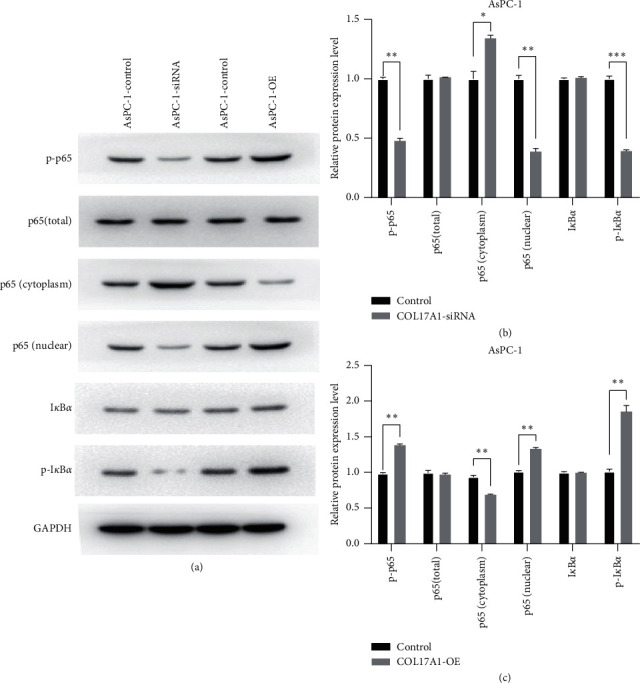
COL17A1 activates NF-kappa B signaling pathway in PDAC cells. (a), (b), and (c) Expressions of p65 (p65 Total), phospho-p65 (p-p65), p65 (cytoplasm), p65 (nuclear), I*κ*B*α*, and phospho-I*κ*B-*α* (p-I*κ*B*α*) protein were determined in siRNA-COL17A1, and overexpression-COL17A1(COL17A1-OE) AsPC-1 cells. Results shown are the mean ± SD (^*∗*^*p* < 0.05, ^*∗∗*^*p* < 0.01, and ^*∗∗∗*^*p* < 0.001).

To further elucidate the role of COL17A1 in signaling pathways, western blotting assays were performed to examine the expression of p65, phospho-p65, p65 (cytoplasm), p65 (nuclear), I*κ*B*α*, and phospho-I*κ*B-*α*. We found that phospho-p65 and phospho-I*κ*B-*α* were noticeably enhanced in cells that were overexpressing COL17A1 but decreased in si-COL17A1 cells. p65 in the nucleus was significantly increased in COL17A1-overexpressing cells but decreased in si-COL17A1 cells (Figures 6(a)–6(c)). These results, thus, showed that COL17A1 may promote the NF-*κ*B signaling pathway activation in apoptosis.

### 3.7. COL17A1 Promotes Tumor Growth In Vivo

**Figure 7:**
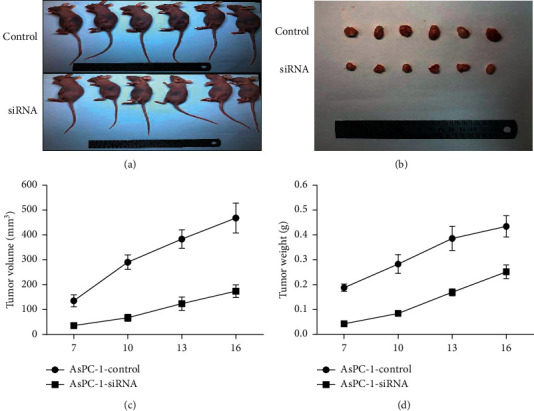
Downregulated COL17A1 reduced the tumor growth in vivo. (a–c) The volume of the tumor xenograft of nude mice in the si-COL17A1 group was smaller than that in the control group. (d) Tumor xenograft growth of nude mice in the si-COL17A1 group was slower when compared to the control group (^*∗*^*p* < 0.05, ^*∗∗*^*p* < 0.01, and ^*∗∗∗*^*p* < 0.001).

To determine the effects of COL17A1 in vivo, AsPC-1 cells were subcutaneously injected into the flanks of nude mice in two groups: the AsPC-1 cells with stable knockdown of COL17A1 gene were injected in the experimental group, and normal AsPC-1 cells were injected in the control group. The tumor growth rates in mice injected with siRNAs-COL17A1 cells were slower than in the control mice (Figures 7(a)–7(d)). Therefore, our data suggest that COL17A1 has a positive effect on the in vivo growth of PDAC cells.

## 4. Discussion

The joint diagnosis of the imaging evaluations and molecular biomarkers cannot entirely monitor the occurrence, progression, and early metastasis of PDAC, so new noninvasive diagnosis strategies are urgently needed. Our data show that the expression of COL17A1 is very high in pancreatic cancer tissues and is closely related to the patients' overall survival time. Stepwise analyses indicate that COL17A1 is an independent prognostic factor for PDAC. However, COL17A1 expression was not significantly correlated with any clinicopathologic factors except the pathology differentiation. The reason for this finding may be limited sample size, in addition to the current treatment strategy and the short overall survival of patients with PDAC. Our results further showed that COL17A1 may promote cell multiplication and attack and inhibit cell apoptosis in PDAC by activating the NF-*κ*B pathway. Collectively, our data suggest that COL17A1 may be a unique biomarker for clinical diagnosis, prognostic assessment, and the therapy of PDAC patients.

Some studies have shown upregulated COL17A1 in ductal breast cancers, prostate carcinoma, cutaneous squamous cell, and basal cell carcinomas [9] and in cervical cancer and squamous cell carcinomas [30–34]. Our results indicate that COL17A1 assumes an oncogene role in many cancers. It is well known that the initial stage of the progression of epithelial cancer characterizes the failure in adhesion of cells and the persistent biological function of related adhesion proteins [35], and this may be related to the type I hemidesmosomes (HDs), which is composed of COL17. Our results further show that the increased expression of COL17A1 in PDAC had a positive correlation with increased cell invasion and progression. A recent study showed that a novel PTEN-COL17A1 fusion gene is involved in regulating COL17 expression and glioblastoma multiforme (GBM) aggressiveness and determined that COL17A1 may play the roles of a prognostic biomarker and potential therapeutic targets of GBM [36]. We, therefore, speculate that COL17A1 works through cell surface receptors to mediate tumor progression as against the function of cell-matrix adhesion molecules in PDAC. COL17A1 and other three adhesion molecules (BPAG1, CDHF7, and CD97) show downregulation mainly between the premalignant and stage II cells and between the normal myoepithelial and the premalignant cells in breast cancer [37]; therefore, we guess that the stage-specificity of COL17A1 may exist in pancreatic cancer because of limited samples even if our results did not show a significant association between COL17A1 expression and clinical stage.

Cells can activate the apoptosis inhibition pathway by targeting the proapoptosis factors and upregulating the antiapoptosis factors expressions to maintain cell survival [38]. NF-*κ* B plays key and complex roles in cell apoptosis; some studies show that TNF-*α* plays a dual role in apoptosis, those of promoting apoptosis by activating caspase-8 and -10 or suppressing apoptosis by activating NF-*κ* B [39, 40]. IAP (inhibitors of apoptosis) can help the cancer cell survival and proliferation by stimulating their biological activity [41]. The IAP may promote cell survival and tumorigenesis according to the ability of many IAPs to function as ubiquitin-E3 ligases which can regulate NF-*κ*B signaling [42]. The activation of this kind of noncanonical NF-*κ*B signaling also frequently takes place in pancreatic cancers [43]. Gemcitabine in combination with p65 siRNA can control the proliferation of pancreatic cancer cells and induce apoptosis as well, both in vivo and in vitro, and curtail the growth and angiogenesis of tumors in nude mice, and this can be a welcome solution as pancreatic cancer treatment is an important issue all over the world [44]. Recently, studies reported that isodeoxyelephantopin (IDET) may function as an anticarcinogenic via the NF-*κ*B inactivation and the regulation of oncogenic lncRNA expression in breast cancer [45]. In addition, NF-*κ*B may present a unique target for tumor avoidance and therapy in combination with inhibition of transcription factors, such as Stat3 [46]. Our data also indicate that COL17A1 may mediate the inhibition of pancreatic cancer cells apoptosis by activating the NF-*κ*B signaling pathway.

In summary, our study revealed that overexpression of COL17A1 is related to reduced survival in PDAC subjects, and COL17A1 can promote proliferative abilities and invasiveness PDAC putatively suppress apoptosis via the NF-*κ*B pathway. Additionally, COL17A1 may serve as a potential molecular therapeutic target. However, our study has some limitations, like limited samples, validation of therapeutic target, and the need for more detailed mechanisms to hence lay the foundation for further research.

## Figures and Tables

**Table 1:** Clinicopathologic characteristics of 169 PDAC patients.

Patients characteristics	Number of cases (%)
*Age (years)*	
≤60	76 (45.0)
>60	93 (55.0)

*Gender*	
Male	96 (56.8)
Female	73 (43.2)

*Clinical stage*	
I or II	95 (56.2)
III or IV	74 (43.8)

*T classification*	
T1 or T2	38 (22.5)
T3 or T4	131 (77.5)

*N classification*	
N0	98 (58.0)
N1-2	71 (42.0)

*Metastasis*	
No	163 (96.4)
Yes	6 (3.6)

*Pathologic differentiation*	
High (middle or high)	115 (68.0)
Low (No or low)	54 (32.0)

*Vascular invasion*	
No	119 (70.4)
Yes	50 (29.6)

*Neural invasion*	
No	87 (51.5)
Yes	82 (48.5)

*Vital states（at follow-up）*	
Alive	70 (41.4)
Dead	99 (58.6)

*Expression of COL17A1*	
Low expression	63 (37.3)
High expression	106 (62.7)

**Table 2:** Interrelationship between expression of COL17A1 and clinicopathologic traits of pancreatic cancer subjects.

Characteristics	COL17A1 expression		*p* value
	Low or none	High	
*Age*			
≤60	30	46	0.633
>60	33	60	

*Sex*			
Male	37	59	0.749
Female	26	47	

*T classification*			
T1 or T2	18	20	0.182
T3 or T4	45	86	

*N classification*			
N0	56	42	**<0.0001**
N1-2	7	64	

*Metastasis*			
No	63	100	0.135
Yes	0	6	

*Clinical stage*			
I or II	56	39	**<0.0001**
III or IV	7	67	

*Pathology differentiation*			
High (middle or high)	53	62	**<0.01**
Low (no or low)	10	44	

*Vascular invasion*			
No	48	71	0.226
Yes	15	35	

*Neural invasion*			
No	38	49	0.082
Yes	25	57	

Patients were staged in accordance with the 8^th^ Edition of the AJCC *Cancer*'s' TNM Classification. The numbers in bold indicate that the *p* values are significant (*p* < 0.05, the chi-square test).

**Table 3:** Univariate and multivariate Cox regression analyses of prognostic characteristics for overall survival in PDAC patients.

Prognostic characteristics	Univariate analysis			Multivariate analysis		
	*p*	HR	95% CI	*p*	HR	95% CI
Age	**<0.05**	1.517	1.008–2.282	**<0.05**	1.523	1.003–2.313
Gender	0.128	1.370	0.913–2.056	—	—	—
Clinical stage	**<0.05**	1.582	1.063–2.354	0.499	—	—
T classification	**<0.01**	2.281	1.327–3.921	0.081	—	—
N classification	0.068	1.448	0.973–2.156	0.444	—	—
Metastasis	**<0.01**	4.546	1.905–10.845	**<0.05**	2.477	1.014–6.053
Pathologic differentiation	0.916	1.023	0.666–1.573	0.626	—	—
Vascular invasion	0.077	1.504	0.957–2.364	—	—	—
Neural invasion	**<0.001**	2.252	1.492–3.400	**<0.01**	1.936	1.264–2.966
COL17A1	**<0.01**	2.201	1.394–3.475	**<0.01**	1.885	1.177–3.019

HR : hazard ratio; CI : confidence interval. The numbers in bold indicate that the *p* values are significant (*p* < 0.05, Cox regression method: forward LR).

## Data Availability

The data about clinicopathologic characteristics of 169 PDAC patients used to support the findings of this study are available from the corresponding author upon request (e-mail: docyao@hotmail.com).
